# Editorial: Insights in pharmacogenetics and pharmacogenomics: 2023

**DOI:** 10.3389/fphar.2024.1540478

**Published:** 2025-01-03

**Authors:** José A. G. Agúndez, Elena García-Martín

**Affiliations:** Institute of Molecular Pathology Biomarkers, Universidad de Extremadura, Cáceres, Spain

**Keywords:** pharmacogenetics, pharmacogenomics, current challenges, latest discoveries, recent advances, pharmacokinetics

This Research Topic seeks to provide new insights, highlight recent advancements, explore current challenges, and discuss future opportunities in the dynamic field of Pharmacogenetics and Pharmacogenomics, and it is a continuation of a previous Research Topic on the same subject (Agundez and Garcia-Martin, 2022). By reflecting on the progress made over the past year and addressing upcoming obstacles, this collection of articles will inspire researchers and offer valuable guidance for the way forward.

This Research Topic consists of five articles contributed by thirty-five authors. As of the writing of this editorial, the papers in this collection have been downloaded nearly 1,200 times. The Research Topic features two review articles, one focusing on the pharmacogenetics and pharmacogenomics of leukemia therapy, and the other on neurodegenerative disorders. Additionally, the collection includes two original research papers examining the genetic variability of the glucocorticoid pathway and the pharmacokinetics of ezetimibe, along with a research report on the genetic variability of the *NUDT15* and *TPMT* genes.

The review paper by Ahmad et al. explores genetic factors influencing drug response in neurodegenerative disorders like Alzheimer’s, Parkinson’s, Huntington’s, and amyotrophic lateral sclerosis. Traditional treatments often fail to consider genetic variability, leading to inconsistent clinical outcomes for these diseases, highlighting key markers like the *APOE ε4* allele in Alzheimer’s and *CYP2D6* polymorphisms in Parkinson’s. It also discusses advancements in pharmacogenomics tools such as GWAS, NGS, and CRISPR-Cas9, and examines the application of pharmacogenomics in clinical practice, along with challenges related to ethics and data integration.

Another review paper, authored by Kaehler et al. focuses on molecular drug resistance biomarkers in BCR::ABL1-driven chronic myeloid leukemia (CML) and JAK2-driven myeloproliferative neoplasms (MPNs). Resistance to targeted therapies is common for both disorders. Secondary mutations in the BCR::ABL1 kinase domain are a well-known cause of resistance to tyrosine kinase inhibitors (TKIs) in CML, while such mutations are rare in JAK2-driven MPNs. Given the high prevalence and lack of specific therapies in MPNs, identifying critical pathways that sustain inhibitor resistance in MPN models is crucial. The review paper highlights alternative signaling pathways, such as PDGFR, Ras, and PI3K/Akt, activated in both CML and MPNs. Additionally, YBX1 nucleoprotein induction and deubiquitinating enzymes are suggested as potential biomarkers for TKI resistance. Authors conclude that whole exome sequencing of patient samples could aid in identifying resistance markers and developing novel diagnostic tools like liquid biopsies and therapeutic agents to overcome resistance in distinct leukemia subtypes.

The study by Stampar et al. was conducted to assess the relationship between glucocorticoid pathway polymorphisms and treatment response in severe COVID-19 patients. The study group was composed of COVID-19 patients treated with dexamethasone or methylprednisolone, and 14 single nucleotide variations and copy number variations involving the genes *NR3C1, CYP3A4, CYP3A5, GSTP1, GSTM1, GSTT1,* and *ABCB1*. The most salient findings were an association of *CYP3A4* rs35599367, the signature single nucleotide variation that characterizes the allele CYP3A4*22 (https://www.pharmvar.org/gene/CYP3A4), to an increased risk of critical disease, ICU treatment, and worse outcomes, including more frequent therapy switches, longer hospital stays, and prolonged oxygen therapy. *NR3C1* rs6198 was associated with shorter dexamethasone treatment, a higher likelihood of therapy switching, and longer hospitalization. *NR3C1* rs33388 was linked to shorter hospital stays and lower ICU treatment odds. *GSTP1* rs1695 affected hospitalization duration and oxygen supplementation. In sum, this study raises potential pharmacogenomics biomarkers of response to glucocorticoid therapy in COVID-19 therapy.

The manuscript authored by Gonzalez-Iglesias et al. aims to assess the effect of nearly 50 gene variants on ezetimibe pharmacokinetics. The rationale for this study is that gene variations such as *ABCG5*, *ABCG8*, *NPC1L1*, and *UGT1A1* have been suggested to affect its levels, but the effect of other pharmacogenes is not well understood and this information is crucial for the development of clinical pharmacogenomics guidelines (Caudle et al., 2014). In a panel of 96 patients, authors determined ezetimibe pharmacokinetics and variations in a comprehensive gene panel including A*BCB1, ABCC2, CYP1A2, CYP2A6, CYP2B6, CYP2C8, CYP2C9, CYP2C19, CYP2D6, CYP3A4, CYP3A5, SLCO1B1, SLC22A1, UGT1A1, UGT1A3, UGT1A4, UGT1A6, UGT1A8, UGT1A9, UGT2B7, UGT2B10,* and *UGT2B15.* Although no significant associations were found between the gene variants studied and ezetimibe pharmacokinetics, univariate analysis revealed trends linking *ABCB1* and *ABCC2* gene variants with Cmax that deserve further investigation. In addition, the lack of association identified in this study provides relevant information in the development of clinical pharmacogenomics guidelines, because evidence ruling out major associations of genotypes with drug response is of great help in providing recommendations and in harmonizing different guidelines (Abdullah-Koolmees et al., 2020, Agundez et al., 2023) and indicating the lack of evidence to provide recommendations when appropriate [see, for instance (Duarte et al., 2024)].

The study by Perini et al. focuses on single nucleotide variations (SNVs) affecting two major pharmacogenes *TPMT* and *NUDT15*, both involved in the biodisposition of thiopurines. These genes are included in clinical pharmacogenomics guidelines (Pratt et al., 2022), but the allele frequencies in several human populations are unknown. By analyzing four SNVs in three native groups living in reservation areas in Brazil, authors concluded that two common *TPMT* polymorphisms, namely, rs1800460 and rs1142345 are unusually common in one of such populations, named Paiter-Suruí. The risk of developing adverse drug events with intolerance is nearly ten times higher in such a population, as compared to other native groups, and about 3–4 times higher as compared to any other population worldwide analyzed so far.

In sum, this Research Topic addressed findings that hold significant value in advancing the fields of Pharmacogenetics and Pharmacogenomics, ultimately contributing to their implementation into clinical practice.
